# Complete sequence and variability of a new subgroup B nepovirus infecting potato in central Peru

**DOI:** 10.1007/s00705-016-3147-6

**Published:** 2016-11-17

**Authors:** Joao De Souza, Giovanna Müller, Wilmer Perez, Wilmer Cuellar, Jan Kreuze

**Affiliations:** 1 International Potato Center (CIP), Apartado 1558, Lima 12, Peru; 2 Facultad de Ciencias Biológicas, Programa de Biología Molecular, Universidad Nacional Mayor de San Marcos, Lima 11, Peru; 3 International Center for Tropical Agriculture (CIAT), Apartado Aéreo 6713, Cali, Colombia

## Abstract

The complete bipartite genome (RNA1 and RNA2) of a new nepovirus infecting potato was obtained using small RNA sequencing and assembly complemented by Sanger sequencing. Each RNA encodes a single polyprotein, flanked by 5’ and 3’ untranslate regions (UTR) and followed by a poly (A) tail. The putative polyproteins encoded by RNA1 and RNA2 had sets of motifs which are characteristic of viruses in the genus *Nepovirus*. Sequence comparisons using the Pro-Pol region and the coat protein, including phylogenetic analysis of these regions, showed closest relationships with nepoviruses. The data obtained support the taxonomical status of this new virus (putative named Potato virus B, PVB) as a member of the genus Nepovirus, subgroup B.

**Electronic supplementary material:**

The online version of this article (doi:10.1007/s00705-016-3147-6) contains supplementary material, which is available to authorized users.

The genus *Nepovirus* (order *Picornavirales*, family *Secoviridae*, sub-family *Comovirinae*) of plant viruses has been divided into three subgroups (A, B, C) based on the length and packaging of RNA2, serological properties, cleavage site specificity of the proteinase, and phylogenetic analysis of the coat protein sequence [1]. There are few nepoviruses infecting potato. In the subgroup A, there are reports of a calico strain of *Tobacco ring spot virus* (TRSV) [2] and *Potato black ring spot virus* (PBRSV) [3]; however, a recent report [4] confirmed that the calico strain of TRSV, the only strain of TRSV that was reported to infect potato [2], is actually a strain of PBRSV. Accordingly, no reports exist of TRSV infecting potato. In the subgroup C, the only virus reported infecting potato is Potato virus U (PVU) [5], but there is no sequence information available yet that can confirm its classification in subgroup C. In subgroup B, *Tomato black ring virus* (TBRV) has been reported to infect potato, but only in Europe. Infection of TBRV in potatoes is normally only sporadic, and the virus is considered of only minor importance in this crop [6]. In this study, the complete genome of a new nepovirus from subgroup B infecting potato in Peru was determined from native potato plants showing calico-like symptoms and subsequently found to be relatively common in such plants in the central Peruvian highlands. This virus was tentatively named Potato virus B (PVB). Whereas the name PVB has been used before for a potato virus, the virus concerned was soon shown to be a strain of PVX [7, 8].

Leaves of native potato plants that showed calico symptoms were collected during the rainy season (March, 2010) at Quichas (Yanacancha, Daniel A. Carrión, Pasco, Peru) (latitude 10°38’ 10.66” and longitude 76º 10’21.78”; at 4007 m.a.s.l.). Total RNA was extracted from these leaves using Trizol reagent (Invitrogen, Carlsbad, CA) following the manufacturer’s instructions. Small RNAs were isolated of one plant as described previously [9], and then, sent to Fasteris Life Sciences SA (Plan-les-Ouates, Switzerland) for library preparation and sequencing on IlluminaHiseq2000 platform in 50 bp single end read mode [10]. Reads were assembled de-novo using Velvet v0.6.04 [11] and the AssemblyAssembler 1.4 script (Jacob Crawford, Cornell University) with a range of hash lengths from 13 to 25. Contigs were identified by BLASTx against GenBank sequences and those corresponding to nepoviruses were extracted. Sequencing of the small RNA library produced a total of 15,871,227 reads between 21-24 nucleotides (nts). Six contigs with similarity to nepoviruses could be identified after assembly using Velvet. Five contigs corresponded to RNA1 of nepoviruses and three of these could be further assembled into a super-contig corresponding to the 3’-end and the other two contigs were close to the 5’-end of RNA1. The remaining contig corresponded to the almost complete RNA2 sequence. The missing intervening regions of RNA1 and terminal regions of both RNAs were successfully amplified by specifically designed primers and adapter ligation (Table 1), after which the corresponding regions were cloned into pGEM-T Easy vector (Promega, WI, USA) and sequencing (Macrogen-Korea). The 3’ and 5’UTR were confirmed by RNA linker mediated rapid amplification of cDNA ends using the ModBan and BanTwo linkers, respectively (Table 1). Sequences were edited and annotated using Vector NTI v.9 software package (Invitrogen). Phylogenetic and molecular evolutionary analyses were conducted using MEGA5 [12]. The complete sequence of both RNAs were deposited in GenBank (accession no. KX656670 and KX656671).

**Table 1:** Primers and linkers used in this study and the corresponding virus region amplified

*Primer name*	*Sequence* (*5*′*-3*′)	*Amplified region*
NepoWP-5UTR-Rin1*	GGTAAAAAAACAAATTAAGACTATTATTATATTTGC	0 - 261
NepoWP1_Fn	TATGGATAGCGCCATAGTTTTACCTTAT	197 - 486
NepoWP1_Rn	CTGTATAGGCAGAAATGGTGGTTATACC	
Nepo1-WP_Fa	TCTCCCACTTATATCCAAGGTTCTTCC	402 - 1928
Nepo1-WP_Ra	CCGCCACTTTTTGCTGCTCTG	
NepoWP-RdRp-F1	CAGCCACCAAGACGGAACCAGCC	2671 - 4648
NepoWP-RdRp-R1	GGTTCTTTAATTGGTACATTGAC	
NepoWP-F2	CAGATTATATGCTCTGAGTGGG	5384 - 6340
NepoWP-R2	GGCAAGCCACCGACACCAGCTCC	
NepoWP-RdRp-F3	GCAAAGCGTGTCACAGGTATACG	6184 - 7082
NepoWP-3UTR-R3	GGACAAACTTATCTCATCGCCAC	
Nepo1-WP_3UTR-F*	CATTCAGGTCGAAGTTTACAGTTGCC	6797 - 7154
Nepo2-WP_5UTR-R*	CTACCAAGGGCTTCAGCTTTGGC	0 - 285
Nepo2-WP_3UTR-F*	GACGGCCGGACCTTTCTCTCC	4204 - 4527
Modban linker	A5’pp CTG TAG GCA CCA TCA AT/ddC/	
Bantwo linker	ATC GTrA rGrGrC rArCrC rUrGrA rArA	

* Used together with RACE primer

The complete sequences of RNA1 and RNA2 of this putative new virus are 7148 and 4527 nts long excluding the poly(A) tail, respectively. Their predicted genomic organization is similar to that of other members of the genus *Nepovirus*, subgroup B. Both RNAs (Fig. 1A) contained a single open reading frame (ORF). The putative 255.79-kDa (2264 aa) polyprotein (P1) from RNA1 was preceded by a 140 nts 5’-UTR and followed by a 216 nts 3’-UTR. P1 covers 95.01% of the RNA1 coding capacity, higher than other nepoviruses of subgroup B. Based on homology, P1 is predicted to be cleaved by its own viral proteinase into six mature proteins: X1 (48.6 kDa, unknown function), X2 (21.6 kDa, often referred to a putative cofactor by analogy to the related 32 kDa comovirus protein), NTB (66.6 kDa, nucleoside-triphosphate-binding protein or putative helicase), VPg (3.1 kDa, viral genome-linked protein), proteinase (Pro) (24.1 kDa) and RNA-dependent RNA polymerase – RdRp (Pol) (91.6 kDa). The putative dipeptides that serve as cleavage sites, deduced by the similarity of the dipeptide alongside the upstream sequence using nepoviruses from subgroup B previously identified, were R_427_/A_428_, R_617_/S_618_, K_1212_/S_1213_, R_1239_/S_1240_, K_1449_/S_1450_, producing the X1, X2, NTP, VPg, Pro and Pol proteins (Fig. 1A). Re-alignment of the reads of the RNA1 using MAQ confirmed the sequence and was able to call all nts with high quality scores at an average coverage of 1551x from 523,735 aligned reads (3.29% of the total siRNA reads) (Fig. S1a). RNA2 has a single ORF that encodes a polyprotein (P2) similar to subgroup A and B nepoviruses and is cleaved into three domains, whereas that of subgroup C nepoviruses contains four domains [1]. P2 has a predicted molecular weight of 153.31 kDa (1,371 aa), which is preceded by a 214 nts 5’-UTR and followed by a 200 nts 3’-UTR. P2 covers 90.85% of the RNA2 coding capacity, higher than RNA2 of other nepoviruses of subgroup B. They are predicted to be processed into mature protein 2A, the putative movement protein (MP), and the coat protein (CP, 61.4 kDa). Based on comparison to other subgroup B nepoviruses, the site containing the putative cleavage site to separate MP from CP is L_812_/K_813_ (Fig. 1B). Re-aligning siRNAs to the consensus sequence of the RNA2 using MAQ confirmed the sequence, calling all nts at an average coverage of 1,289x by 274,946 reads (1.73% of the total reads) (Fig S1b). The identity between the 3’-UTRs of both RNAs of PVB was 95% (typical for nepoviruses), but the identity between the 5’-UTRs was 38%, less than those of nepoviruses previously reported. Comparison among PVB and other members of the genus *Nepovirus*, show that, PVB has a similarity range with subgroup B nepoviruses reported from 53 - 71% in the Pro-Pol region, and from 25 – 34% in the CP region (Table S1). A phylogenetic tree generated using alignments of these regions located PVB in the subgroup B of the genus *Nepovirus* (Fig. 1C and D). Comparison of the sequence of PVB with the unpublished sequences of two previously reported potato infecting nepoviruses in Peru, Arracacha virus A and PVU, showed no significant homology (Ian Adams & Roger Jones, personal communication), confirming PVB is a newly discovered virus.

**Fig. 1:**
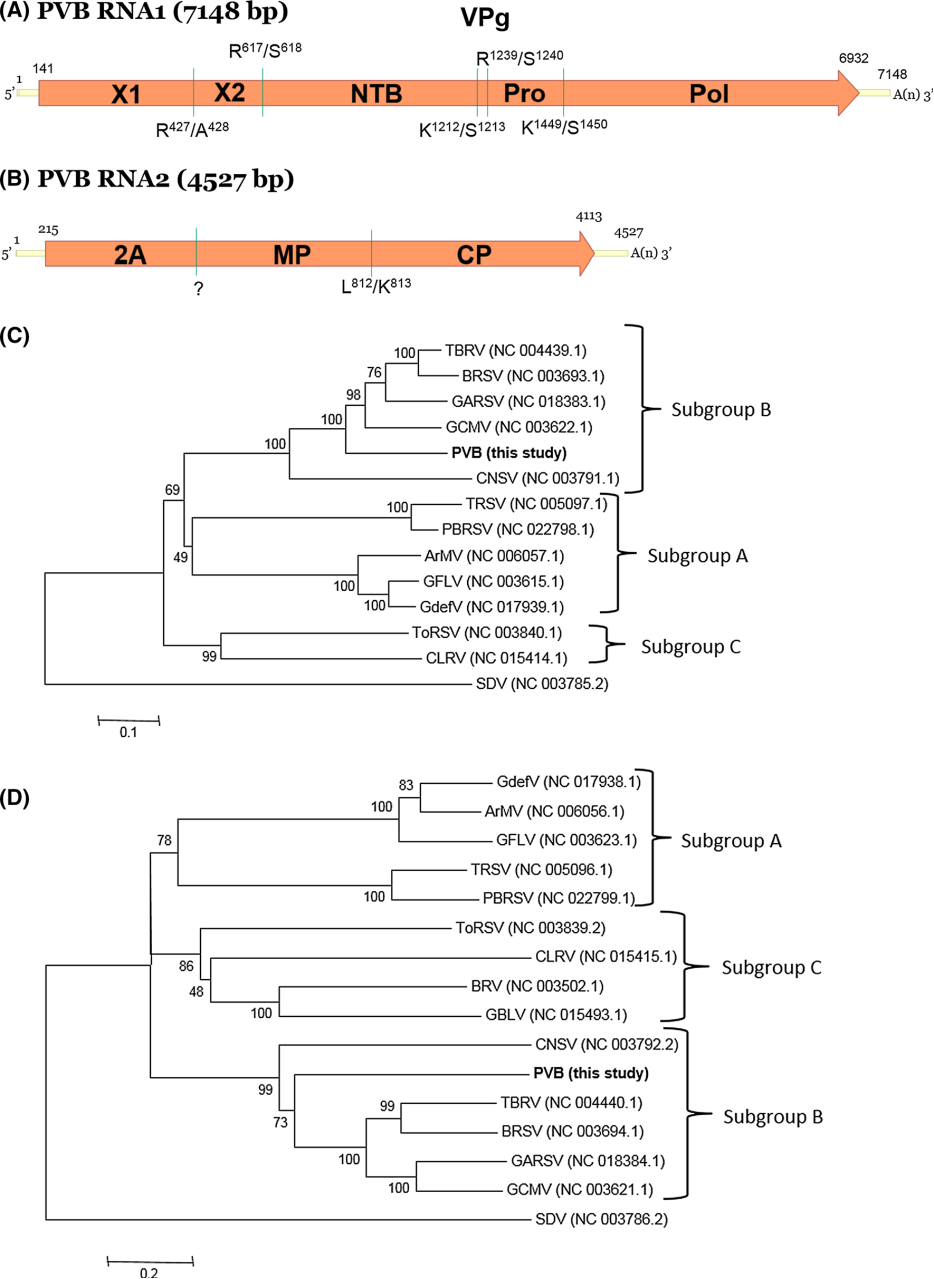
A and B) Genome structure of Potato virus B. Open arrows indicate open reading frames, with the names of predicted gene products inside them. Polyprotein cleavage sites that release the specific proteins are indicated by vertical green lines. Question mark in RNA2 means that we were unable to identify the exact putative cleavage site to separate 2A from MP and its location is an estimation. C and D) Phylogenetic tree based on an alignment of the Pro-Pol and CP region respectively. Sequences were aligned using the ClustalW algorithm, and the tree was generated using the neighbour-joining method. Numbers on branches indicate percentage of bootstrap support out of 1000 bootstrap replications. *Satsuma dwarf virus* (SDV) was used as outgroup. TBRV (*Tomato black ring virus*), BRSV (*Beet ringspot virus*), GARSV (*Grapevine Anatolian ringspot* *virus*), GCMV (*Grapevine chrome mosaic virus*), CNSV (*Cycas necrotic stunt virus*), TRSV (*Tobacco ringspot virus*). PBRSV (*Potato black ringspot virus*), ArMV (*Arabis mosaic virus*), GFLV (*Grapevine fanleaf virus*), GdefV (*Grapevine deformation virus*), ToRSV (*Tomato ringspot virus*), CLRV (*Chery leaf roll virus*), BRV (*Blackcurrant reversion virus*), GBLV (*Grapevine Bulgarian Latent virus*)

Primers, that amplify the polymerase region and were used to confirm the genome obtained, were also used to detect this virus in leaves of additional potato plants with calico symptoms collected from Pasco and Junín (departments in Peru) (Figure S2). From 151 samples from Pasco, 28 were positive (19%); however, out of 67 samples evaluated from Junín, only two were positive (3%) (Table S2). The amplified 1,977 bp RdRp fragment of some positive samples were also sequenced to evaluate the PVB variability. In the phylogenetic tree produced with these sequences, two groups could be distinguished (86.8% average nt identity between the two groups and >98.7% or 95.7% identity within the two groups respectively), but there seemed to be little geographic relationship (Fig. S3)

The sequence similarity found, the organization genome and the phylogenetic analysis indicated that PVB corresponds to a new virus species belonging to the genus *Nepovirus* within subgroup B, according to [1]. Many nepoviruses are transmitted by soil-inhabiting nematodes belonging to three closely related genera *Xiphinema*, *Longidorus*, or *Paralongidorus* in the order *Dorylaimida*, family *Londigoridae* [13]. *Longidorus* is so far the only nematode genus reported to transmit subgroup B nepoviruses, whereas the genus *Xiphinema* and *Paralongidorus* were reported to transmit subgroup A and C nepoviruses. Genus *Longidorus* is considered a quarantine species by the National Agriculture Health Service (SENASA, its acronym Spanish) [14]. However Jones *et al.* [5] reported preliminary experiments with *Longidorus* spp., which had been collected in Peru (Cesar Fribourg, personal communication) and Ciancio et al [15, 16] also reported the presence of longidorid nematodes in Peru. It would be a priority to confirm what the vector of PVB is and confirm the presence of *Longidorus* spp. in Peru. The phylogenetic tree using the symptomatic plants sampled from Pasco and Huancayo show that variability is present in this virus, similar to GFLV as determined by Oliver *et al.* [17] where RdRp also was used as a source to analyze virus variability. On the other hand, whereas PVB was always found associated with calico like symptoms (all positive samples had calico symptoms), not all sampled plants with these symptoms tested positive for the virus. Thus it remains unclear to what extent PVB contributes to the observed symptoms. Future studies into this aspect, including tentative yield impacts should be an additional priority.

## Electronic supplementary material

Below is the link to the electronic supplementary material.

Fig. S1 Line graph showing sequencing coverage and depth over the a) RNA1 and b) RNA2 of PVB by siRNA reads of positive (above the horizontal line, positive values) and negative (below the horizontal line, negative values) sense. X axis represents nucleotide position and Y axis represents the fold sequence coverage (frequency) of each nucleotide position (PPTX 410 kb)

Fig. S2 Location of fields evaluated. (Purple dots indicate fields where virus was detected and red dots indicate fields where no virus was detected) (PPTX 859 kb)

Fig. S3 Phylogenetic tree of PVB isolates constructed from polymerase region amplified showing 2 putative groups. PVB_RNA1 is the sample analyzed by deep sequencing (PPTX 36 kb)

Supplementary material 4 (DOCX 19 kb)
