# Supplementary figures and images for: Complete sequence and variability of a new subgroup B nepovirus infecting potato in central Peru
Source: Arch Virol. 2016 Nov 17;162(3):885–9. doi: 10.1007/s00705-016-3147-6 (PMC5329089; doi:10.1007/s00705-016-3147-6)

## Slide 1
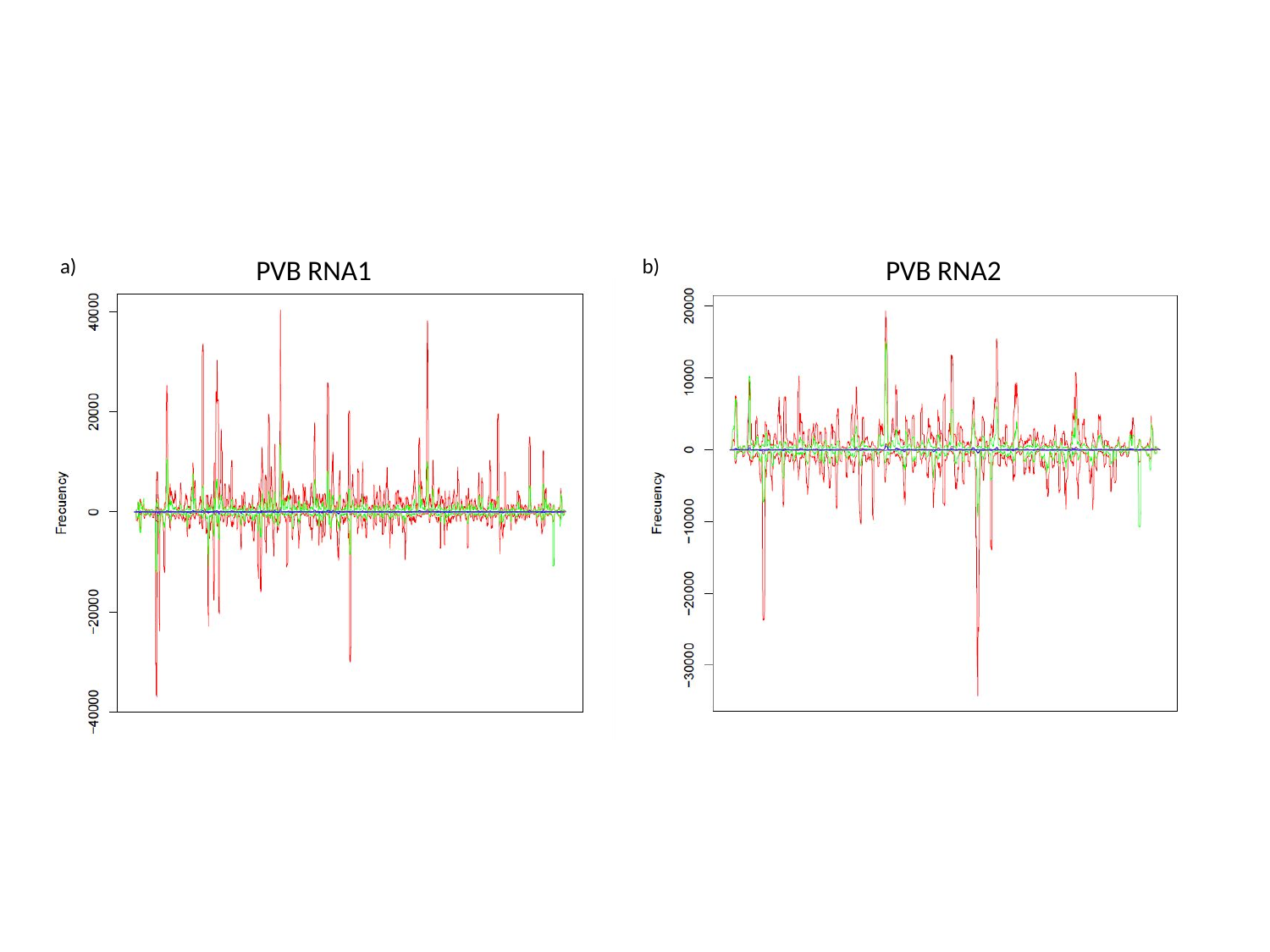

PVB RNA1
a)
b)
PVB RNA2

Supplement: Supplementary file 1 — Fig. S1 Line graph showing sequencing coverage and depth over the a) RNA1 and b) RNA2 of PVB by siRNA reads of positive (above the horizontal line, positive values) and negative (below the horizontal line, negative values) sense. X axis represents nucleotide position and Y axis represents the fold sequence coverage (frequency) of each nucleotide position (PPTX 410 kb) [file 705_2016_3147_MOESM1_ESM.pptx]

## Slide 1
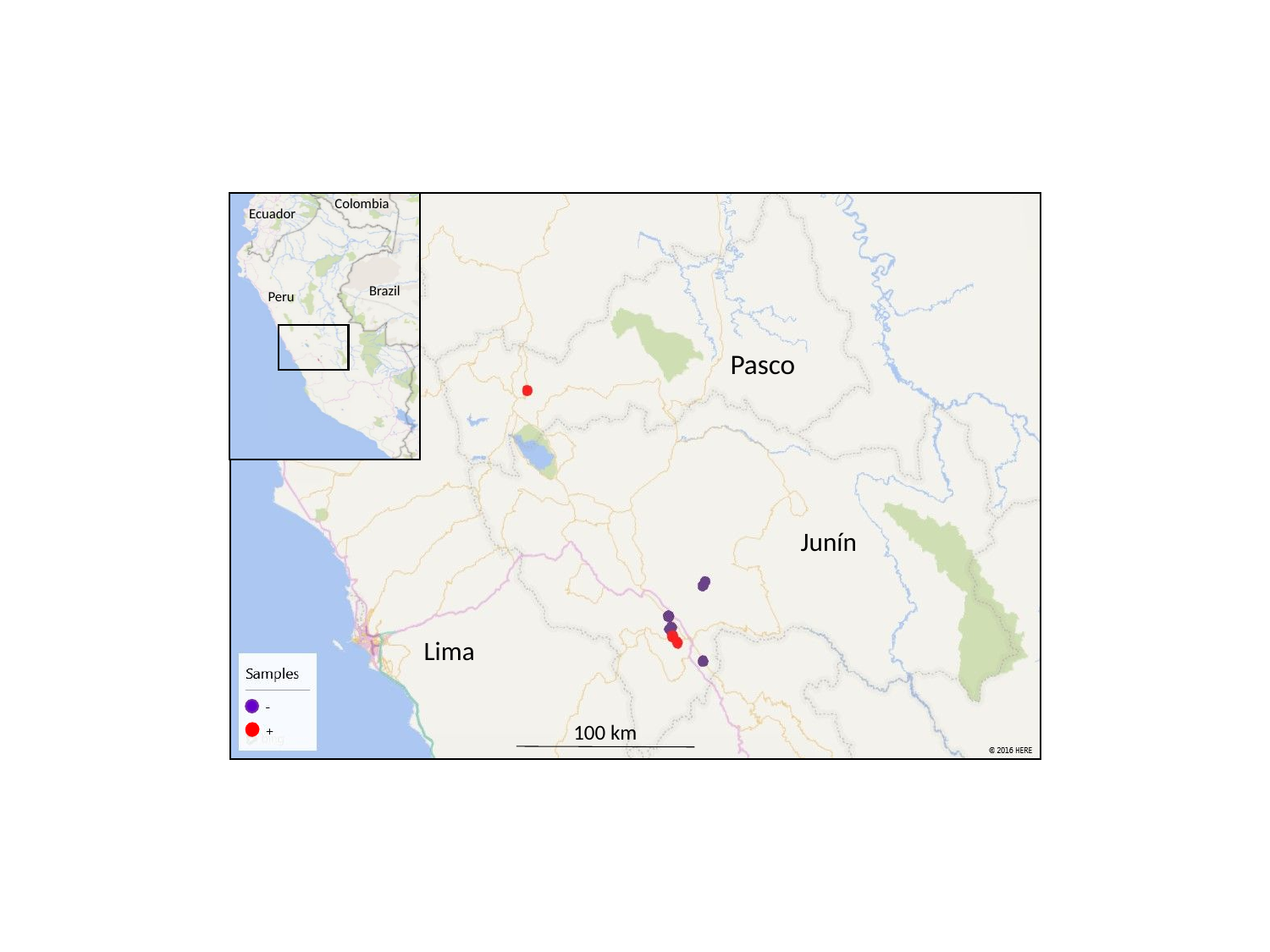

Colombia
Ecuador
Brazil
Peru
Pasco
Junín
Lima
100 km

Supplement: Supplementary file 2 — Fig. S2 Location of fields evaluated. (Purple dots indicate fields where virus was detected and red dots indicate fields where no virus was detected) (PPTX 859 kb) [file 705_2016_3147_MOESM2_ESM.pptx]
