# Supplementary material for: Complete sequence and variability of a new subgroup B nepovirus infecting potato in central Peru
Source: Arch Virol. 2016 Nov 17;162(3):885–9. doi: 10.1007/s00705-016-3147-6 (PMC5329089; doi:10.1007/s00705-016-3147-6)
Supplement: Supplementary file 3 — Fig. S3 Phylogenetic tree of PVB isolates constructed from polymerase region amplified showing 2 putative groups. PVB_RNA1 is the sample analyzed by deep sequencing (PPTX 36 kb) [file 705_2016_3147_MOESM3_ESM.pptx]

## Slide 1
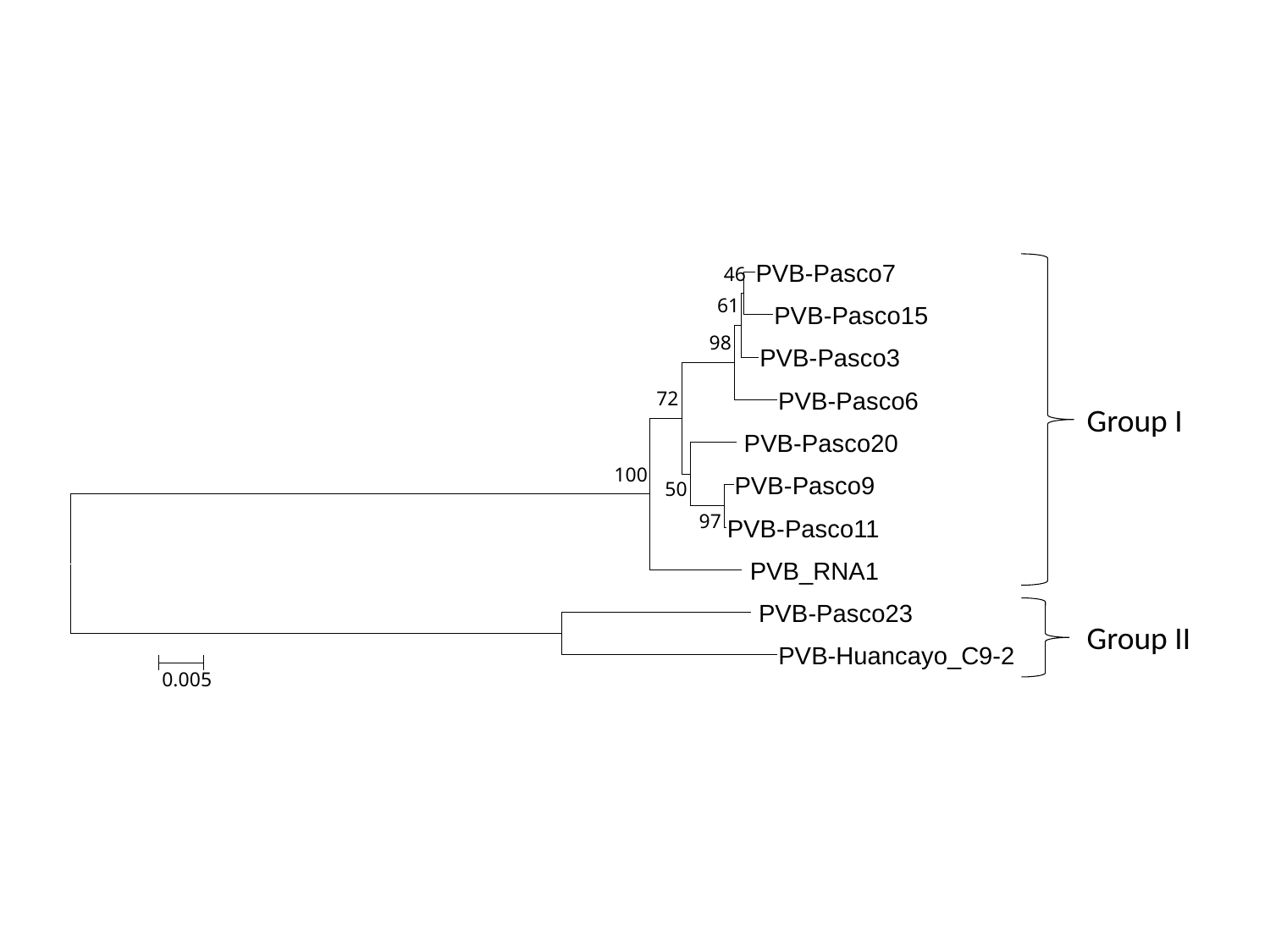

PVB-Pasco7
46
61
PVB-Pasco15
98
PVB-Pasco3
PVB-Pasco6
72
 PVB-Pasco20
100
PVB-Pasco9
50
97
PVB-Pasco11
 PVB_RNA1
 PVB-Pasco23
PVB-Huancayo_C9-2
0.005
Group I
Group II
