# Supplementary material for: Complete sequence and variability of a new subgroup B nepovirus infecting potato in central Peru
Source: Arch Virol. 2016 Nov 17;162(3):885–9. doi: 10.1007/s00705-016-3147-6 (PMC5329089; doi:10.1007/s00705-016-3147-6)
Supplement: Supplementary file 4 — Supplementary material 4 (DOCX 19 kb) [file 705_2016_3147_MOESM4_ESM.docx]

Table S1: Amino acid sequence identity matrix (in percentages) of the Pro-Pol and CP regions Potato calico associated virus with the corresponding sequences of the most closely related viruses [Tomato black ring virus (TBRV), Grapevine chrome mosaic virus (GCMV), Beet ringspot virus (BRSV), Cycas necrotic stunt virus (CNSV), Grapevine Anatolian ringspot virus (GARSV)].

| **PVB** | **Pro-Pol** | **CP** |
| --- | --- | --- |
| TBRV | 71 | 32 |
| GCMV | 68 | 30 |
| BRSV | 70 | 31 |
| CNSV | 53 | 25 |
| GARSV | 69 | 34 |

Table S2: Data on the locations of the samples evaluated and the number of positive PVB samples per site.

| **Department** | **Location** | **Altitude** | **Latitude** | **Longitude** | **Samples** | **PVB** |
| --- | --- | --- | --- | --- | --- | --- |
| Pasco | Quichas | 4007 | -10.65444444 | -76.17638889 | 151 | 28 |
| Junín | Mucchu | 3824 | -11.76527778 | -75.14555556 | 8 | 0 |
| Junín | Mucchu | 3824 | -11.76055556 | -75.14 | 3 | 0 |
| Junín | Pomamanta | 3579 | -11.73972222 | -75.13 | 5 | 0 |
| Junín | San Antonio de Sicaya | 3354 | -11.98833333 | -75.32666667 | 8 | 0 |
| Junín | San Antonio de Sicaya | 3378 | -11.99972222 | -75.33527778 | 5 | 0 |
| Junín | Pucará | 3327 | -12.16888889 | -75.14833333 | 7 | 0 |
| Junín | Chupaca | 3264 | -12.0725 | -75.29138889 | 10 | 1 |
| Junín | Huayao | 3334 | -12.03861111 | -75.32055556 | 10 | 1 |
| Junín | Mitos | 3279 | -11.92833333 | -75.34138889 | 11 | 0 |
